# 9-(Pyrrolidinium-1-yl)-9-boranuidabi­cyclo­[3.3.1]nona­ne

**DOI:** 10.1107/S2414314623003322

**Published:** 2023-04-18

**Authors:** Marcus Herbig, Uwe Böhme

**Affiliations:** a Institut für Anorganische Chemie, Technische Universität Bergakademie Freiberg, Leipziger Str. 29, 09599 Freiberg, Germany; University Koblenz-Landau, Germany

**Keywords:** crystal structure

## Abstract

The title compound, C_12_H_24_BN, is an adduct formed from 9-borabi­cyclo­[3.3.1]nonane (9-BBN) and pyrrolidine. It crystallizes in the triclinic space group *P*  with three mol­ecules in the asymmetric unit, one of which has disorder of the pyrrolidine ring. The B—N bond lengths are between 1.631 (2) and 1.641 (2) Å. The boron and nitro­gen atoms are bound to one hydrogen atom each. These hydrogen atoms are in anti­periplanar orientation. Both six-membered rings of the 9-BBN unit are in a chair conformation in all three mol­ecules. Differences between the three crystallographic independent mol­ecules are found in the five-membered rings of the pyrrolidine unit. These adopt different twisted and envelope conformations.

## Abstract 2

The title compound, C_12_H_24_BN, is an adduct formed from 9-borabi­cyclo­[3.3.1]nonane (9-BBN) and pyrrolidine. It crystallizes in the the triclinic space group *P* 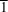 with three mol­ecules in the asymmetric unit.

## Graphical abstract

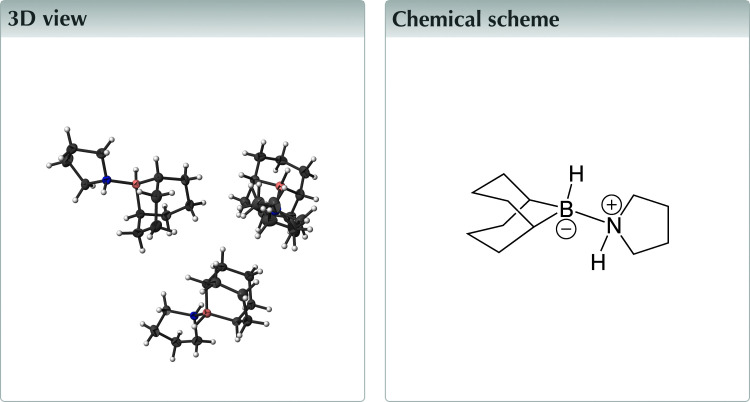

## Structure description

Hydro­boration has been proved to be a powerful tool in organic chemistry (Brown, 1961 ▸). 9-Borabi­cyclo­[3.3.1]nonane (9-BBN) has found extensive use among the various hydro­borating reagents because of its unique properties, commercial availability, convenient preparation, and enormous synthetic applications (Dhillon, 2007 ▸). The present work describes the synthesis and crystal structure of an adduct formed from 9-borabi­cyclo­[3.3.1]nonane and pyrrolidine.

There are three closely related structures that contain the 9-BBN unit bound to an amine, *viz.* *N*-[9-borabi­cyclo­(3.3.1)non­yl]quinuclidine (Yalpani *et al.*, 1988 ▸), 2,6-di­cyclo­hexyl-3,3:5,5-bis­(1,5-*cyclo*-octa­ndi­yl)-2,5-aza­zonia-4-hydro­nia-3,5-diboratin (Boese *et al.*, 1994 ▸), and 9-(di­methyl­amino)[9-borabi­cyclo­(3.3.1)nonan-9-yl]-9-borabi­cyclo­(3.3.1)non­ane (Metzler & Nöth, 1995 ▸). The latter two are dimeric structures.

**Figure 1:**
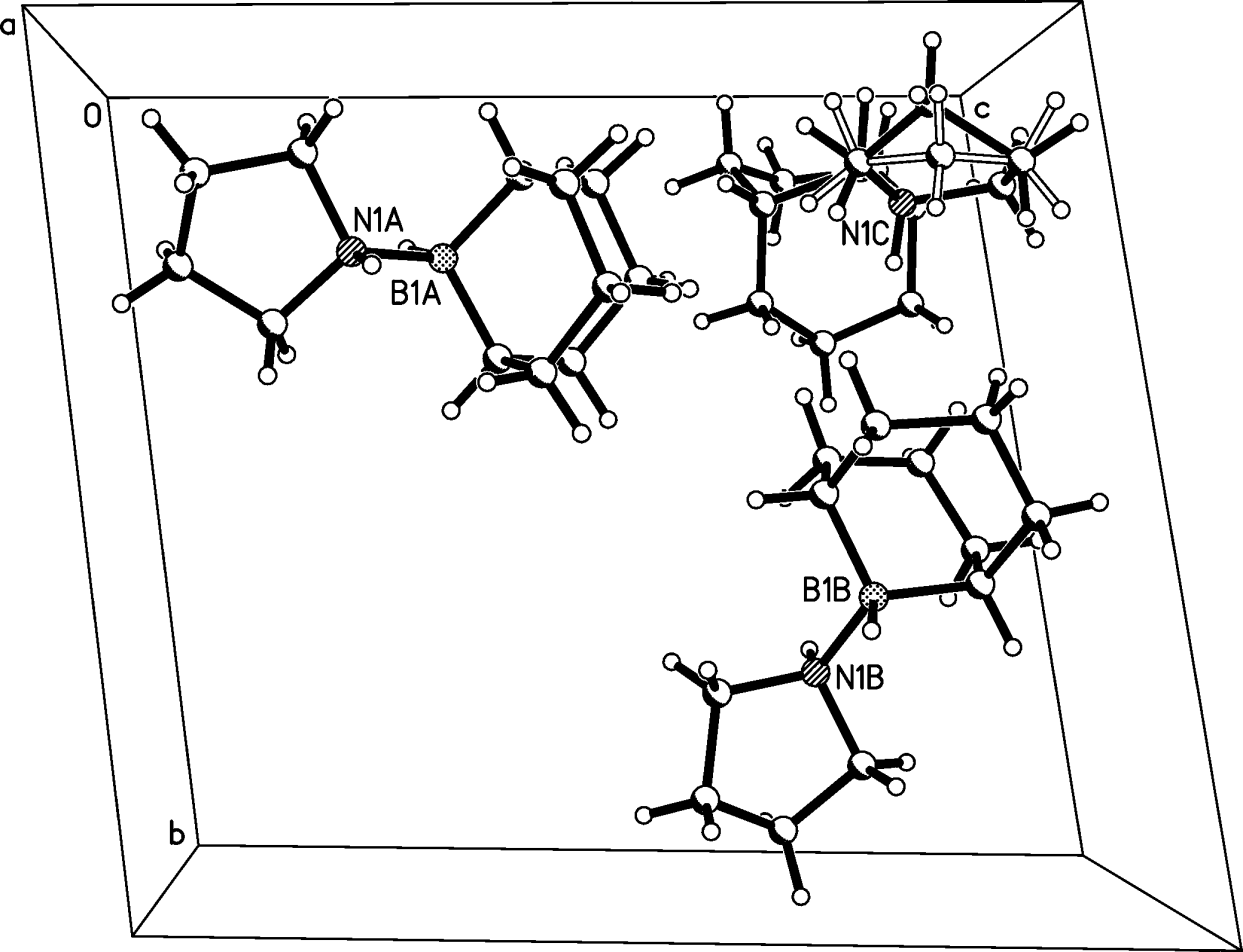
The asymmetric unit of the crystal structure in the unit cell.

The title compound crystallizes in the triclinic space group *P* 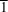 with three mol­ecules in the asymmetric unit (see Figs. 1 ▸–4 ▸ ▸ ▸). On first sight, all three mol­ecules look very similar. The B—N bond lengths are 1.632 (2), 1.631 (2), and 1.641 (2) Å in mol­ecules *A*, *B*, and *C* respectively. The sum of the covalent radii for nitro­gen (0.74 Å) and boron (0.81 Å) is 1.55 Å if one includes the Schomaker–Stevenson correction for partially ionic single covalent bonds (Pauling, 1962 ▸; Schomaker & Stevenson, 1941 ▸). The B—N bonds found in the title compound are longer, which might be explained with the adduct character of the compound under investigation, which formally consists of *R*_2_BH^−^–NH*R*_2_^+^. Indeed, the closely related *N*-(9-borabi­cyclo­(3.3.1)non­yl)quinuclidine has a B—N bond length of 1.676 (3) Å.

The boron and nitro­gen atoms are bound to one hydrogen atom each. These hydrogen atoms are in an anti­periplanar orientation in all three mol­ecules.

**Figure 5:**
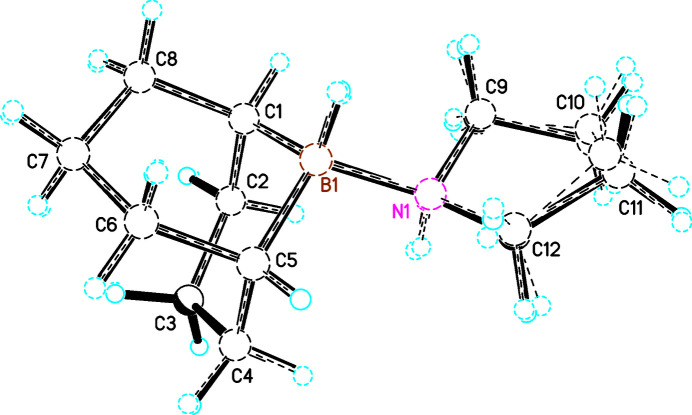
Overlay between the three crystallographic independent mol­ecules obtained by fitting the 9-BBN units.

The 9-BBN unit has a unique geometry imposed by the catenation of the atoms in the bicyclic heterocycles. Both six-membered rings B1/C1–C5 and B1/C5–C8/C1 are in a chair conformation in all three mol­ecules. Differences between the three crystallographic independent mol­ecules become visible with a closer inspection of the five-membered N1/C9–C12 rings. The conformational analysis was performed with the *PLATON* software (Spek, 2009 ▸, 2020 ▸). The five-membered ring in mol­ecule *A* is an envelope on C10*A* and twisted on C9*B*—C10*B* in mol­ecule *B*. Mol­ecule *C* has disorder at the C11 atom of the ring with site-occupation factors of 0.723 (8)/0.277 (8). Therefore, two ring conformations result here. The ring N1*C*/C9*C*–C12*C* is twisted on C10*C*—C11*C*. The ring N1*C*—C9*C*—C10*C*—C11*D*—C12*C* is twisted on C12*C*—N1*C* (Evans & Boeyens, 1989 ▸). Fig. 5 ▸ shows a mol­ecule-fitting plot of all three crystallographically independent mol­ecules in order to visualize these differences. The fitting of the 9-BBN units is perfect, whereas the pyrrolidine rings show small differences.

Inter­molecular inter­actions are dominated by close-packing. No specific hydrogen bonds can be identified.

## Synthesis and crystallization

1.34 g 9-BBN (11 mmol, synthesized from BH_3_·SMe_2_ and 1,5-cyclo­octa­diene) were suspended in 5 ml of toluene (VWR Analapuran, dried with MBRAUN SPS 800) and 1.75 g pyrrolidine (25 mmol, Sigma-Aldrich, distilled from sodium) were added. A gas evolved and the 9-BBN dissolved in the solvent. After standing overnight all volatiles were removed *in vacuo*. After recrystallization from CHCl_3_ 1.15 g of white crystals were obtained, which were used for further analyses and crystal structure analysis.

Yield: 54%; m.p. 339 K (decomp.).

^11^B NMR (CDCl_3_, 160 MHz, δ p.p.m.) −1.50 (*d*, *J* = 95.4 Hz); ^1^H NMR (CDCl_3_, 500 MHz; the spectrum is difficult to inter­pret, because of many overlapping signals) 0.74 (1*H*, *br*, B–H), 1.32–2.05 (18*H*, *m*, 9-BBN unit and pyrrolidine C10—H and C11—H), 2.71–3.33 (4*H*, *m*, pyrrolidine C9—H and C12—H), 3.8 (1*H*, *br*, N–H); ^13^C NMR (CDCl_3_, 125 MHz, δ p.p.m.) 22.3 (B—CH), 24.4 (**C**H_2_CH_2_N), 24.7 and 25.8 (**C**H_2_CHB), 30.2 and 34.4 (**C**H_2_CH_2_CHB), 49.5 and 49.6 (NCH_2_) p.p.m..

IR (KBr, cm^−1^) 3232.3 (*vw*), 2971.9 (*w*), 2912.1 (*m*), 2836.9 (*vs*), 2821.5 (*vs*), 2682.6 (*w*), 2657.6 (*w*), 2217.8 (*w*), 2158.1 (*w*), 2127.2 (*w*), 1620.0 (*w*), 1600.7 (*vw*), 1512.0 (*w*), 1483.1 (*m*), 1454.1 (*m*), 1378.9 (*w*), 1365.4 (*w*), 1344.2 (*w*), 1305.6 (*m*), 1280.6 (*m*), 1269.0 (*m*), 1243.9 (*m*), 1232.4 (*m*), 1205.4 (*vs*), 1155.2 (*s*), 1091.6 (*s*), 1068.4 (*s*), 1041.4 (*vs*), 1008.6 (*w*), 964.3 (*w*), 945.0 (*m*), 912.2 (*vs*), 896.8 (*vs*), 875.6 (*m*), 813.9 (*w*), 756.0 (*s*), 729.0 (*vs*), 694.3 (*m*), 640.3 (*m*), 624.9 (*m*), 605.6 (*w*).

## Refinement

Crystal data, data collection and structure refinement details are summarized in Table 1 ▸.

## Supplementary Material

Crystal structure: contains datablock(s) I. DOI: 10.1107/S2414314623003322/im4018sup1.cif

Structure factors: contains datablock(s) I. DOI: 10.1107/S2414314623003322/im4018Isup2.hkl

Supporting information file. DOI: 10.1107/S2414314623003322/im4018Isup3.cml

CCDC reference: 2255508

Additional supporting information:  crystallographic information; 3D view; checkCIF report

## Figures and Tables

**Figure 2:**
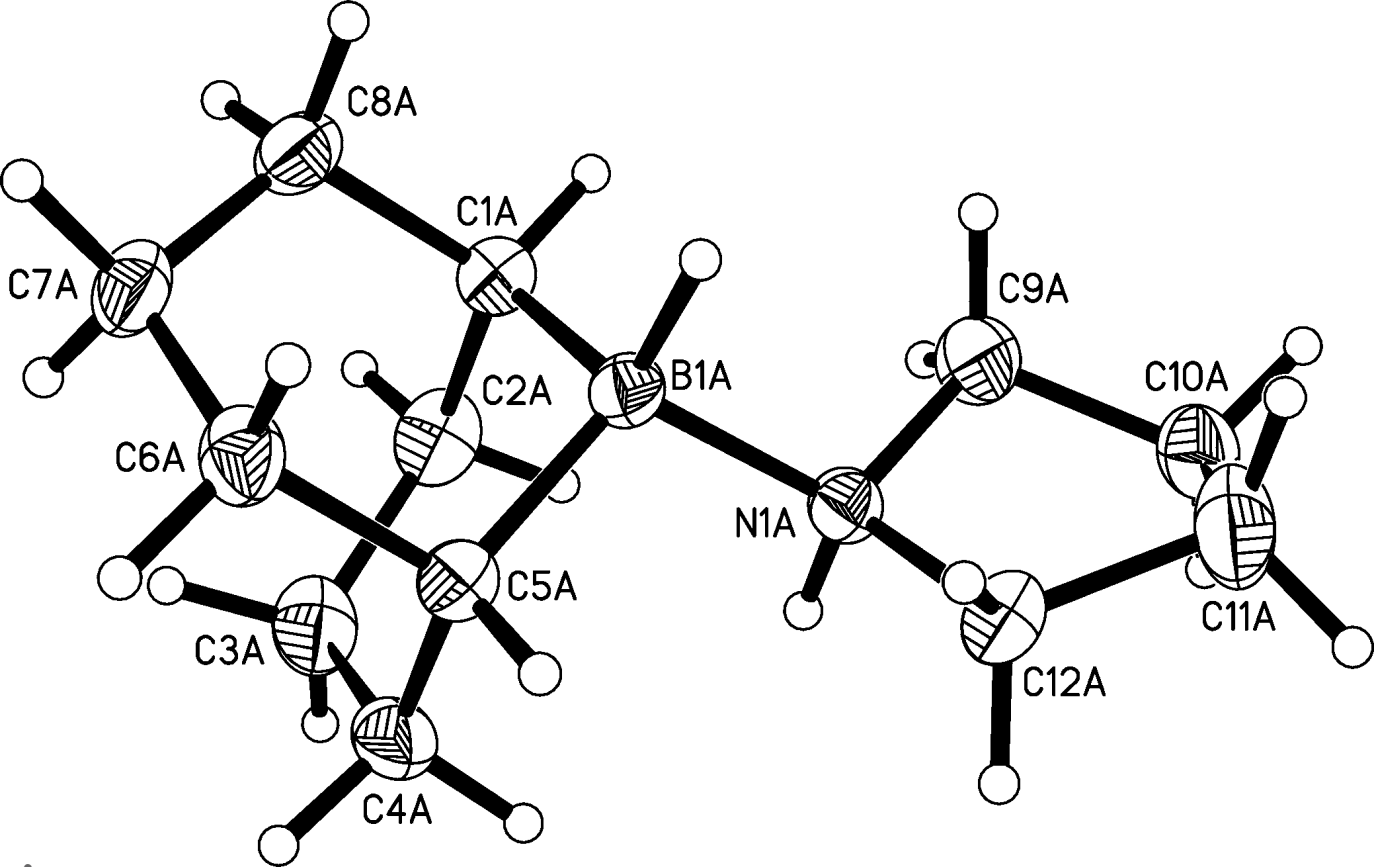
Diagram of mol­ecule *A* showing the atom-labelling scheme. Atomic displacement parameters are drawn at the 50% probability level.

**Figure 3:**
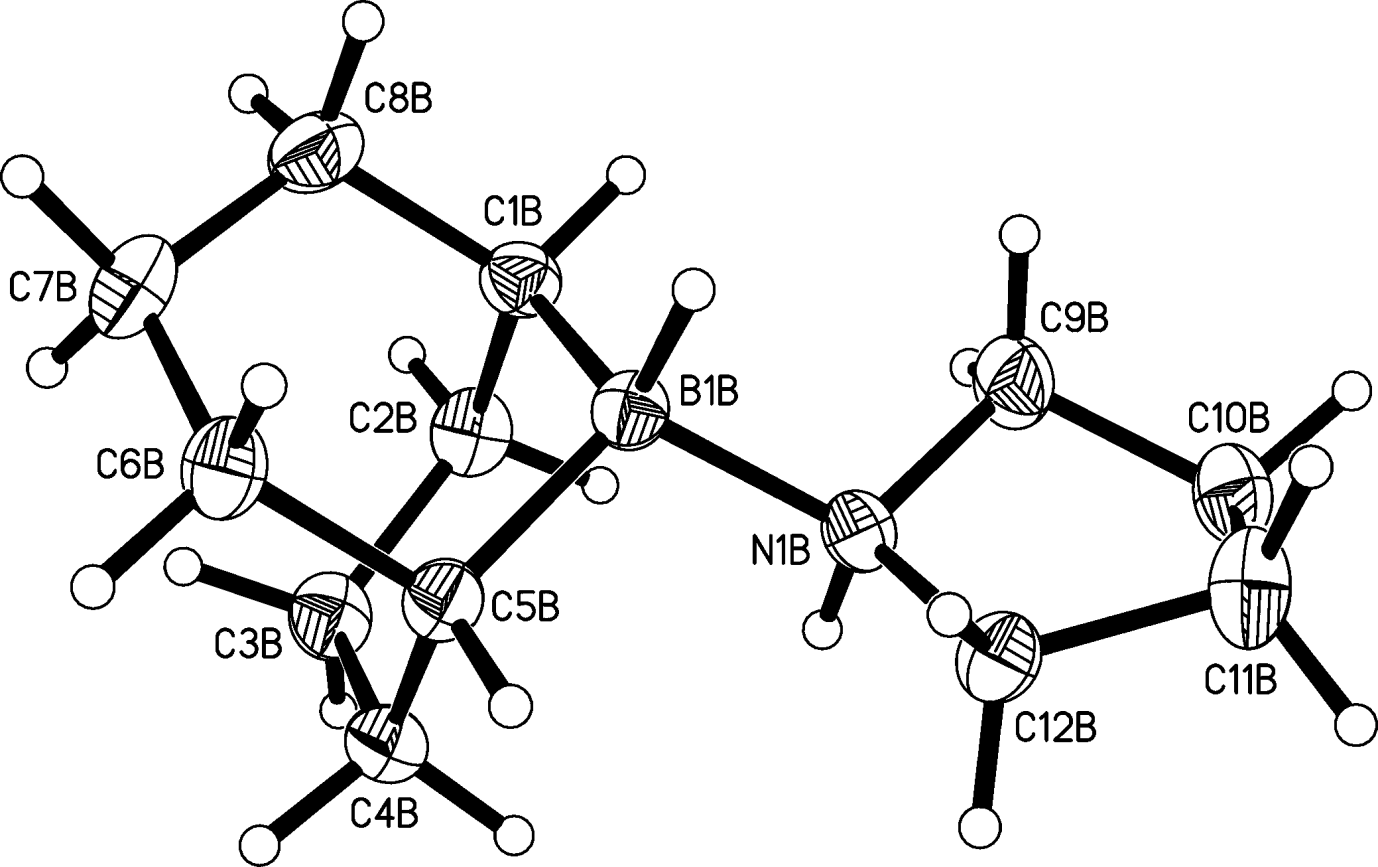
Diagram of mol­ecule *B* showing the atom-labelling scheme. Atomic displacement parameters are drawn at the 50% probability level.

**Figure 4:**
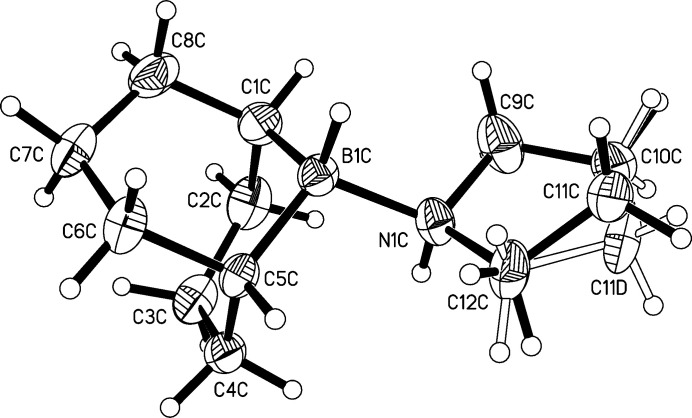
Diagram of mol­ecule *C* showing the atom-labelling scheme. Atomic displacement parameters are drawn at the 50% probability level.

**Table 1:** Experimental details

Crystal data	
Chemical formula	C_12_H_24_BN
*M*_r_	193.13
Crystal system, space group	Triclinic, *P* 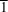
Temperature (K)	153
*a*, *b*, *c* (Å)	10.0432 (5), 12.7045 (6), 14.3846 (7)
α, β, γ (°)	81.305 (4), 84.313 (4), 82.068 (4)
*V* (Å^3^)	1791.19 (15)
*Z*	6
Radiation type	Mo *K*α
μ (mm^−1^)	0.06
Crystal size (mm)	0.45 × 0.45 × 0.40

Data collection	
Diffractometer	Stoe *IPDS* 2T
Absorption correction	Integration (*X-RED*; Stoe, 2009 ▸)
*T*_min_, *T*_max_	0.806, 0.931
No. of measured, independent and observed [*I* > 2σ(*I*)] reflections	33005, 8226, 6579
*R*_int_	0.043
(sin θ/λ)_max_ (Å^−1^)	0.650

Refinement	
*R*[*F* ^2^ > 2σ(*F* ^2^)], *wR*(*F* ^2^), *S*	0.043, 0.115, 1.04
No. of reflections	8226
No. of parameters	413
No. of restraints	3
H-atom treatment	H atoms treated by a mixture of independent and constrained refinement
Δρ_max_, Δρ_min_ (e Å^−3^)	0.32, −0.20

Computer programs: *X-AREA* and *X-RED* (Stoe, 2009 ▸), SHELXT (Sheldrick, 2015*a* ▸), *SHELXL2017/1* (Sheldrick, 2015*b* ▸) and *ORTEP-3 for Windows* (Farrugia, 2012 ▸).

## full crystallographic data

### Crystal data

**Table:**

C_12_H_24_BN	*Z* = 6
*M**_r_* = 193.13	*F*(000) = 648
Triclinic, *P*1	*D*_x_ = 1.074 Mg m^−^^3^
*a* = 10.0432 (5) Å	Mo *K*α radiation, λ = 0.71073 Å
*b* = 12.7045 (6) Å	Cell parameters from 33005 reflections
*c* = 14.3846 (7) Å	θ = 2.7–27.9°
α = 81.305 (4)°	µ = 0.06 mm^−^^1^
β = 84.313 (4)°	*T* = 153 K
γ = 82.068 (4)°	Prism, colourless
*V* = 1791.19 (15) Å^3^	0.45 × 0.45 × 0.40 mm

### Data collection

**Table:**

Stoe IPDS 2T diffractometer	8226 independent reflections
Radiation source: sealed X-ray tube, 12 x 0.4 mm long-fine focus	6579 reflections with *I* > 2σ(*I*)
Plane graphite monochromator	*R*_int_ = 0.043
Detector resolution: 6.67 pixels mm^-1^	θ_max_ = 27.5°, θ_min_ = 2.9°
rotation method scans	*h* = −13→13
Absorption correction: integration (X-RED; Stoe, 2009)	*k* = −16→16
*T*_min_ = 0.806, *T*_max_ = 0.931	*l* = −18→18
33005 measured reflections	

### Refinement

**Table:**

Refinement on *F*^2^	Primary atom site location: structure-invariant direct methods
Least-squares matrix: full	Hydrogen site location: mixed
*R*[*F*^2^ > 2σ(*F*^2^)] = 0.043	H atoms treated by a mixture of independent and constrained refinement
*wR*(*F*^2^) = 0.115	*w* = 1/[σ^2^(*F*_o_^2^) + (0.0446*P*)^2^ + 0.5868*P*] where *P* = (*F*_o_^2^ + 2*F*_c_^2^)/3
*S* = 1.04	(Δ/σ)_max_ < 0.001
8226 reflections	Δρ_max_ = 0.32 e Å^−^^3^
413 parameters	Δρ_min_ = −0.20 e Å^−^^3^
3 restraints	

### Special details

**Table:**

Geometry. All esds (except the esd in the dihedral angle between two l.s. planes) are estimated using the full covariance matrix. The cell esds are taken into account individually in the estimation of esds in distances, angles and torsion angles; correlations between esds in cell parameters are only used when they are defined by crystal symmetry. An approximate (isotropic) treatment of cell esds is used for estimating esds involving l.s. planes.
Refinement. Hydrogen atoms bonded to C were positioned geometrically and allowed to ride on their parent atoms, with C—H = 0.99 Å for CH_2_ and 1.0 Å for CH (C1 and C5). *U*_iso_(H) = *xU*_eq_(C), where *x* = 1.2 for CH_2_ and CH. Hydrogen atoms at nitrogen and boron were localized from residual electron density maps. Hydrogen atoms at nitrogen were freely refined. Hydrogen atoms at boron were restrained at a B–H distance of 1.08 Å (*DFIX* restraint in *SHELXL*).

### Fractional atomic coordinates and isotropic or equivalent isotropic displacement parameters (Å^2^)

**Table:**

	*x*	*y*	*z*	*U*_iso_*/*U*_eq_	Occ. (<1)
B1A	0.79459 (12)	0.26768 (10)	0.36908 (9)	0.0229 (2)	
H1D	0.7013 (12)	0.2510 (10)	0.3343 (9)	0.022 (3)*	
N1A	0.91179 (10)	0.26727 (8)	0.28198 (7)	0.0237 (2)	
H1N	0.9888 (17)	0.2866 (13)	0.3007 (11)	0.040 (4)*	
C1A	0.83681 (12)	0.17621 (10)	0.45594 (8)	0.0269 (2)	
H1A	0.852259	0.104907	0.432630	0.032*	
C2A	0.96854 (13)	0.19650 (11)	0.49325 (9)	0.0345 (3)	
H2A	0.987338	0.143090	0.549519	0.041*	
H2B	1.043479	0.184205	0.444419	0.041*	
C3A	0.96769 (13)	0.30920 (12)	0.51990 (10)	0.0373 (3)	
H3A	0.915953	0.312970	0.581828	0.045*	
H3B	1.061549	0.320328	0.527150	0.045*	
C4A	0.90690 (13)	0.40062 (11)	0.44809 (9)	0.0335 (3)	
H4A	0.974870	0.413217	0.394207	0.040*	
H4B	0.888561	0.466901	0.478058	0.040*	
C5A	0.77558 (12)	0.38105 (9)	0.40966 (8)	0.0252 (2)	
H5A	0.752784	0.439665	0.356872	0.030*	
C6A	0.65506 (12)	0.38055 (10)	0.48518 (9)	0.0296 (3)	
H6A	0.646594	0.447471	0.513902	0.035*	
H6B	0.571681	0.381336	0.453556	0.035*	
C7A	0.66454 (12)	0.28469 (11)	0.56439 (8)	0.0312 (3)	
H7A	0.724752	0.298474	0.610492	0.037*	
H7B	0.573882	0.280191	0.597575	0.037*	
C8A	0.71689 (13)	0.17584 (10)	0.53178 (9)	0.0314 (3)	
H8A	0.641628	0.148969	0.506730	0.038*	
H8B	0.744197	0.124450	0.587515	0.038*	
C9A	0.95264 (15)	0.16064 (10)	0.24822 (9)	0.0355 (3)	
H9A	0.874771	0.119349	0.254335	0.043*	
H9B	1.024984	0.118110	0.284924	0.043*	
C10A	1.00278 (15)	0.18694 (12)	0.14554 (10)	0.0394 (3)	
H10A	0.999353	0.126437	0.110160	0.047*	
H10B	1.096013	0.205724	0.139111	0.047*	
C11A	0.90267 (17)	0.28319 (14)	0.11258 (10)	0.0458 (4)	
H11A	0.941365	0.326970	0.056141	0.055*	
H11B	0.818327	0.259923	0.097177	0.055*	
C12A	0.87631 (13)	0.34629 (10)	0.19567 (8)	0.0298 (3)	
H12A	0.933039	0.405423	0.186867	0.036*	
H12B	0.780390	0.377232	0.202174	0.036*	
B1B	0.70440 (12)	0.64836 (10)	0.74750 (9)	0.0219 (2)	
H1E	0.8054 (13)	0.6822 (11)	0.7314 (10)	0.032 (4)*	
N1B	0.60317 (10)	0.73847 (7)	0.68679 (7)	0.02243 (19)	
H2N	0.5214 (16)	0.7158 (12)	0.6902 (10)	0.030 (4)*	
C1B	0.65523 (12)	0.63456 (9)	0.85877 (8)	0.0251 (2)	
H1B	0.650576	0.705773	0.881320	0.030*	
C2B	0.51348 (12)	0.59802 (10)	0.87556 (8)	0.0284 (2)	
H2C	0.488533	0.586334	0.944326	0.034*	
H2D	0.447829	0.656461	0.847091	0.034*	
C3B	0.50149 (12)	0.49525 (10)	0.83492 (9)	0.0298 (3)	
H3C	0.404789	0.487016	0.835625	0.036*	
H3D	0.544338	0.433019	0.876378	0.036*	
C4B	0.56666 (12)	0.49307 (10)	0.73374 (9)	0.0276 (2)	
H4C	0.505040	0.537969	0.689733	0.033*	
H4D	0.575024	0.418534	0.719376	0.033*	
C5B	0.70668 (11)	0.53243 (9)	0.71410 (8)	0.0231 (2)	
H5B	0.731702	0.539234	0.644611	0.028*	
C6B	0.81818 (12)	0.45578 (10)	0.76475 (9)	0.0291 (2)	
H6C	0.818033	0.383103	0.747826	0.035*	
H6D	0.906521	0.479653	0.740788	0.035*	
C7B	0.80494 (13)	0.44822 (11)	0.87269 (9)	0.0338 (3)	
H7C	0.892469	0.415486	0.896628	0.041*	
H7D	0.737329	0.399330	0.898297	0.041*	
C8B	0.76362 (13)	0.55556 (11)	0.91104 (8)	0.0315 (3)	
H8C	0.845285	0.591741	0.909041	0.038*	
H8D	0.729810	0.540130	0.978085	0.038*	
C9B	0.57846 (14)	0.84566 (10)	0.72116 (9)	0.0325 (3)	
H9C	0.661133	0.863101	0.744526	0.039*	
H9D	0.505256	0.846814	0.772569	0.039*	
C10B	0.53817 (13)	0.92408 (10)	0.63525 (9)	0.0322 (3)	
H10C	0.551062	0.998251	0.642301	0.039*	
H10D	0.443077	0.922347	0.623502	0.039*	
C11B	0.63473 (13)	0.88215 (10)	0.55673 (10)	0.0351 (3)	
H11C	0.722706	0.909798	0.553954	0.042*	
H11D	0.596635	0.903587	0.494781	0.042*	
C12B	0.65039 (12)	0.76050 (9)	0.58322 (8)	0.0266 (2)	
H12C	0.595027	0.727962	0.544742	0.032*	
H12D	0.745849	0.729956	0.572356	0.032*	
B1C	0.36310 (13)	0.12899 (11)	0.83426 (10)	0.0270 (3)	
H1F	0.3975 (16)	0.0402 (10)	0.8482 (11)	0.038 (4)*	
N1C	0.49310 (11)	0.18179 (9)	0.85926 (8)	0.0305 (2)	
H3N	0.4766 (17)	0.2531 (14)	0.8425 (12)	0.043 (4)*	
C1C	0.22926 (13)	0.16740 (11)	0.89924 (9)	0.0338 (3)	
H1C	0.246900	0.144008	0.966932	0.041*	
C2C	0.19823 (14)	0.29047 (12)	0.88339 (11)	0.0395 (3)	
H2E	0.112921	0.311254	0.920635	0.047*	
H2F	0.270691	0.320984	0.908317	0.047*	
C3C	0.18534 (13)	0.34108 (10)	0.78081 (11)	0.0376 (3)	
H3E	0.189223	0.419155	0.776240	0.045*	
H3F	0.095714	0.331479	0.762630	0.045*	
C4C	0.29416 (13)	0.29474 (10)	0.70996 (9)	0.0318 (3)	
H4E	0.377344	0.327714	0.712113	0.038*	
H4F	0.263388	0.316074	0.645738	0.038*	
C5C	0.32941 (11)	0.17170 (9)	0.72638 (8)	0.0258 (2)	
H5C	0.411241	0.151893	0.684025	0.031*	
C6C	0.21508 (13)	0.11162 (11)	0.70492 (10)	0.0345 (3)	
H6E	0.190968	0.138402	0.639712	0.041*	
H6F	0.249448	0.034441	0.707534	0.041*	
C7C	0.08666 (13)	0.12307 (11)	0.77209 (11)	0.0370 (3)	
H7E	0.035897	0.194793	0.754879	0.044*	
H7F	0.029053	0.068757	0.763048	0.044*	
C8C	0.11333 (14)	0.10963 (12)	0.87666 (11)	0.0413 (3)	
H8E	0.133818	0.032062	0.899353	0.050*	
H8F	0.029595	0.136577	0.912633	0.050*	
C9C	0.51960 (16)	0.16283 (15)	0.96171 (10)	0.0459 (4)	
H9E	0.479559	0.225471	0.992894	0.055*	
H9F	0.479981	0.098613	0.994241	0.055*	
C10C	0.66937 (17)	0.14639 (16)	0.96501 (12)	0.0564 (5)	
H10E	0.696875	0.103756	1.025055	0.068*	0.723 (8)
H10F	0.707400	0.215440	0.955605	0.068*	0.723 (8)
H10G	0.698566	0.205445	0.992597	0.068*	0.277 (8)
H10H	0.697222	0.077892	1.005031	0.068*	0.277 (8)
C11C	0.7120 (2)	0.0813 (3)	0.87756 (17)	0.0442 (9)	0.723 (8)
H11E	0.808648	0.082195	0.856224	0.053*	0.723 (8)
H11F	0.692351	0.006088	0.892810	0.053*	0.723 (8)
C11D	0.7260 (5)	0.1444 (8)	0.8787 (5)	0.044 (2)	0.277 (8)
H11G	0.793037	0.079580	0.877775	0.053*	0.277 (8)
H11H	0.774570	0.207952	0.860122	0.053*	0.277 (8)
C12C	0.62421 (13)	0.14377 (13)	0.80686 (10)	0.0403 (3)	
H12E	0.607972	0.098329	0.759986	0.048*	0.723 (8)
H12F	0.667289	0.205850	0.773273	0.048*	0.723 (8)
H12G	0.642940	0.193432	0.748471	0.048*	0.277 (8)
H12H	0.623940	0.070785	0.790682	0.048*	0.277 (8)

### Atomic displacement parameters (Å^2^)

**Table:**

	*U*^11^	*U*^22^	*U*^33^	*U*^12^	*U*^13^	*U*^23^
B1A	0.0199 (6)	0.0270 (6)	0.0223 (6)	−0.0059 (5)	0.0002 (4)	−0.0042 (5)
N1A	0.0226 (5)	0.0256 (5)	0.0235 (5)	−0.0044 (4)	−0.0003 (4)	−0.0055 (4)
C1A	0.0277 (6)	0.0269 (6)	0.0258 (5)	−0.0046 (4)	−0.0009 (4)	−0.0028 (4)
C2A	0.0256 (6)	0.0448 (7)	0.0312 (6)	0.0002 (5)	−0.0055 (5)	−0.0009 (5)
C3A	0.0240 (6)	0.0589 (9)	0.0333 (6)	−0.0110 (6)	−0.0044 (5)	−0.0142 (6)
C4A	0.0299 (6)	0.0388 (7)	0.0365 (7)	−0.0147 (5)	0.0045 (5)	−0.0154 (5)
C5A	0.0248 (5)	0.0261 (5)	0.0249 (5)	−0.0050 (4)	0.0007 (4)	−0.0040 (4)
C6A	0.0241 (6)	0.0357 (6)	0.0292 (6)	−0.0016 (5)	0.0011 (4)	−0.0094 (5)
C7A	0.0249 (6)	0.0446 (7)	0.0243 (6)	−0.0078 (5)	0.0035 (4)	−0.0058 (5)
C8A	0.0319 (6)	0.0355 (6)	0.0262 (6)	−0.0107 (5)	0.0002 (5)	0.0014 (5)
C9A	0.0421 (7)	0.0282 (6)	0.0356 (7)	−0.0033 (5)	0.0077 (5)	−0.0107 (5)
C10A	0.0431 (8)	0.0434 (8)	0.0343 (7)	−0.0102 (6)	0.0093 (6)	−0.0176 (6)
C11A	0.0515 (9)	0.0615 (10)	0.0264 (6)	−0.0073 (7)	−0.0042 (6)	−0.0122 (6)
C12A	0.0291 (6)	0.0344 (6)	0.0243 (6)	−0.0043 (5)	0.0008 (4)	−0.0002 (5)
B1B	0.0194 (5)	0.0229 (6)	0.0230 (6)	−0.0026 (4)	−0.0015 (4)	−0.0018 (5)
N1B	0.0221 (5)	0.0202 (4)	0.0245 (5)	−0.0027 (4)	0.0008 (4)	−0.0034 (4)
C1B	0.0273 (6)	0.0257 (5)	0.0233 (5)	−0.0043 (4)	−0.0014 (4)	−0.0057 (4)
C2B	0.0266 (6)	0.0313 (6)	0.0252 (5)	−0.0025 (5)	0.0044 (4)	−0.0025 (5)
C3B	0.0248 (6)	0.0304 (6)	0.0333 (6)	−0.0075 (5)	−0.0001 (5)	0.0000 (5)
C4B	0.0288 (6)	0.0251 (6)	0.0305 (6)	−0.0057 (4)	−0.0059 (5)	−0.0045 (5)
C5B	0.0237 (5)	0.0236 (5)	0.0212 (5)	−0.0006 (4)	−0.0013 (4)	−0.0034 (4)
C6B	0.0257 (6)	0.0263 (6)	0.0329 (6)	0.0039 (4)	−0.0023 (5)	−0.0031 (5)
C7B	0.0304 (6)	0.0354 (7)	0.0320 (6)	0.0033 (5)	−0.0078 (5)	0.0037 (5)
C8B	0.0325 (6)	0.0390 (7)	0.0236 (6)	−0.0065 (5)	−0.0068 (5)	−0.0014 (5)
C9B	0.0422 (7)	0.0223 (6)	0.0319 (6)	0.0023 (5)	−0.0032 (5)	−0.0060 (5)
C10B	0.0322 (6)	0.0231 (6)	0.0391 (7)	0.0007 (5)	−0.0021 (5)	−0.0013 (5)
C11B	0.0312 (6)	0.0296 (6)	0.0388 (7)	−0.0005 (5)	0.0040 (5)	0.0062 (5)
C12B	0.0278 (6)	0.0281 (6)	0.0228 (5)	−0.0030 (4)	−0.0006 (4)	−0.0012 (4)
B1C	0.0237 (6)	0.0255 (6)	0.0309 (7)	−0.0012 (5)	−0.0008 (5)	−0.0040 (5)
N1C	0.0264 (5)	0.0347 (6)	0.0302 (5)	0.0028 (4)	−0.0053 (4)	−0.0079 (4)
C1C	0.0278 (6)	0.0417 (7)	0.0297 (6)	−0.0025 (5)	0.0036 (5)	−0.0040 (5)
C2C	0.0277 (6)	0.0441 (8)	0.0481 (8)	0.0019 (5)	0.0054 (6)	−0.0224 (6)
C3C	0.0279 (6)	0.0238 (6)	0.0601 (9)	0.0014 (5)	−0.0021 (6)	−0.0077 (6)
C4C	0.0264 (6)	0.0296 (6)	0.0375 (7)	−0.0028 (5)	−0.0034 (5)	0.0015 (5)
C5C	0.0194 (5)	0.0279 (6)	0.0303 (6)	−0.0020 (4)	0.0015 (4)	−0.0078 (5)
C6C	0.0248 (6)	0.0387 (7)	0.0436 (7)	−0.0048 (5)	−0.0018 (5)	−0.0168 (6)
C7C	0.0241 (6)	0.0340 (7)	0.0551 (8)	−0.0076 (5)	0.0010 (5)	−0.0127 (6)
C8C	0.0280 (7)	0.0437 (8)	0.0492 (8)	−0.0090 (6)	0.0085 (6)	−0.0005 (6)
C9C	0.0433 (8)	0.0642 (10)	0.0300 (7)	0.0089 (7)	−0.0091 (6)	−0.0154 (7)
C10C	0.0501 (9)	0.0769 (12)	0.0440 (9)	−0.0300 (9)	−0.0238 (7)	0.0195 (8)
C11C	0.0258 (10)	0.0500 (19)	0.0503 (13)	0.0014 (10)	0.0009 (8)	0.0061 (11)
C11D	0.021 (2)	0.050 (5)	0.067 (4)	−0.003 (2)	−0.008 (2)	−0.022 (3)
C12C	0.0225 (6)	0.0581 (9)	0.0417 (7)	−0.0008 (6)	−0.0026 (5)	−0.0147 (7)

### Geometric parameters (Å, º)

**Table:**

B1A—C5A	1.6148 (17)	C7B—C8B	1.5370 (19)
B1A—C1A	1.6191 (17)	C7B—H7C	0.9900
B1A—N1A	1.6319 (15)	C7B—H7D	0.9900
B1A—H1D	1.163 (11)	C8B—H8C	0.9900
N1A—C9A	1.4985 (15)	C8B—H8D	0.9900
N1A—C12A	1.5127 (15)	C9B—C10B	1.5163 (17)
N1A—H1N	0.919 (17)	C9B—H9C	0.9900
C1A—C8A	1.5428 (16)	C9B—H9D	0.9900
C1A—C2A	1.5431 (17)	C10B—C11B	1.5239 (18)
C1A—H1A	1.0000	C10B—H10C	0.9900
C2A—C3A	1.536 (2)	C10B—H10D	0.9900
C2A—H2A	0.9900	C11B—C12B	1.5248 (17)
C2A—H2B	0.9900	C11B—H11C	0.9900
C3A—C4A	1.535 (2)	C11B—H11D	0.9900
C3A—H3A	0.9900	C12B—H12C	0.9900
C3A—H3B	0.9900	C12B—H12D	0.9900
C4A—C5A	1.5438 (17)	B1C—C5C	1.6173 (18)
C4A—H4A	0.9900	B1C—C1C	1.6207 (18)
C4A—H4B	0.9900	B1C—N1C	1.6406 (17)
C5A—C6A	1.5442 (16)	B1C—H1F	1.127 (12)
C5A—H5A	1.0000	N1C—C9C	1.5013 (17)
C6A—C7A	1.5364 (18)	N1C—C12C	1.5077 (17)
C6A—H6A	0.9900	N1C—H3N	0.899 (18)
C6A—H6B	0.9900	C1C—C2C	1.538 (2)
C7A—C8A	1.5347 (19)	C1C—C8C	1.5436 (19)
C7A—H7A	0.9900	C1C—H1C	1.0000
C7A—H7B	0.9900	C2C—C3C	1.528 (2)
C8A—H8A	0.9900	C2C—H2E	0.9900
C8A—H8B	0.9900	C2C—H2F	0.9900
C9A—C10A	1.5154 (18)	C3C—C4C	1.5372 (19)
C9A—H9A	0.9900	C3C—H3E	0.9900
C9A—H9B	0.9900	C3C—H3F	0.9900
C10A—C11A	1.520 (2)	C4C—C5C	1.5417 (17)
C10A—H10A	0.9900	C4C—H4E	0.9900
C10A—H10B	0.9900	C4C—H4F	0.9900
C11A—C12A	1.5197 (19)	C5C—C6C	1.5427 (16)
C11A—H11A	0.9900	C5C—H5C	1.0000
C11A—H11B	0.9900	C6C—C7C	1.5368 (18)
C12A—H12A	0.9900	C6C—H6E	0.9900
C12A—H12B	0.9900	C6C—H6F	0.9900
B1B—C5B	1.6134 (17)	C7C—C8C	1.534 (2)
B1B—C1B	1.6182 (16)	C7C—H7E	0.9900
B1B—N1B	1.6308 (15)	C7C—H7F	0.9900
B1B—H1E	1.145 (12)	C8C—H8E	0.9900
N1B—C9B	1.4988 (15)	C8C—H8F	0.9900
N1B—C12B	1.5144 (14)	C9C—C10C	1.494 (2)
N1B—H2N	0.902 (15)	C9C—H9E	0.9900
C1B—C2B	1.5430 (16)	C9C—H9F	0.9900
C1B—C8B	1.5461 (17)	C10C—C11D	1.316 (7)
C1B—H1B	1.0000	C10C—C11C	1.600 (4)
C2B—C3B	1.5326 (18)	C10C—H10E	0.9900
C2B—H2C	0.9900	C10C—H10F	0.9900
C2B—H2D	0.9900	C10C—H10G	0.9900
C3B—C4B	1.5382 (17)	C10C—H10H	0.9900
C3B—H3C	0.9900	C11C—C12C	1.477 (3)
C3B—H3D	0.9900	C11C—H11E	0.9900
C4B—C5B	1.5427 (16)	C11C—H11F	0.9900
C4B—H4C	0.9900	C11D—C12C	1.525 (6)
C4B—H4D	0.9900	C11D—H11G	0.9900
C5B—C6B	1.5424 (15)	C11D—H11H	0.9900
C5B—H5B	1.0000	C12C—H12E	0.9900
C6B—C7B	1.5348 (18)	C12C—H12F	0.9900
C6B—H6C	0.9900	C12C—H12G	0.9900
C6B—H6D	0.9900	C12C—H12H	0.9900
			
C5A—B1A—C1A	106.44 (9)	C6B—C7B—H7D	108.5
C5A—B1A—N1A	110.22 (9)	C8B—C7B—H7D	108.5
C1A—B1A—N1A	110.77 (9)	H7C—C7B—H7D	107.5
C5A—B1A—H1D	114.2 (7)	C7B—C8B—C1B	116.04 (10)
C1A—B1A—H1D	112.7 (6)	C7B—C8B—H8C	108.3
N1A—B1A—H1D	102.5 (7)	C1B—C8B—H8C	108.3
C9A—N1A—C12A	106.20 (9)	C7B—C8B—H8D	108.3
C9A—N1A—B1A	114.85 (9)	C1B—C8B—H8D	108.3
C12A—N1A—B1A	114.01 (9)	H8C—C8B—H8D	107.4
C9A—N1A—H1N	105.9 (10)	N1B—C9B—C10B	104.44 (10)
C12A—N1A—H1N	105.8 (10)	N1B—C9B—H9C	110.9
B1A—N1A—H1N	109.4 (10)	C10B—C9B—H9C	110.9
C8A—C1A—C2A	113.04 (10)	N1B—C9B—H9D	110.9
C8A—C1A—B1A	107.59 (10)	C10B—C9B—H9D	110.9
C2A—C1A—B1A	110.52 (9)	H9C—C9B—H9D	108.9
C8A—C1A—H1A	108.5	C9B—C10B—C11B	102.28 (10)
C2A—C1A—H1A	108.5	C9B—C10B—H10C	111.3
B1A—C1A—H1A	108.5	C11B—C10B—H10C	111.3
C3A—C2A—C1A	115.10 (11)	C9B—C10B—H10D	111.3
C3A—C2A—H2A	108.5	C11B—C10B—H10D	111.3
C1A—C2A—H2A	108.5	H10C—C10B—H10D	109.2
C3A—C2A—H2B	108.5	C10B—C11B—C12B	104.44 (10)
C1A—C2A—H2B	108.5	C10B—C11B—H11C	110.9
H2A—C2A—H2B	107.5	C12B—C11B—H11C	110.9
C4A—C3A—C2A	114.34 (10)	C10B—C11B—H11D	110.9
C4A—C3A—H3A	108.7	C12B—C11B—H11D	110.9
C2A—C3A—H3A	108.7	H11C—C11B—H11D	108.9
C4A—C3A—H3B	108.7	N1B—C12B—C11B	106.66 (10)
C2A—C3A—H3B	108.7	N1B—C12B—H12C	110.4
H3A—C3A—H3B	107.6	C11B—C12B—H12C	110.4
C3A—C4A—C5A	115.16 (10)	N1B—C12B—H12D	110.4
C3A—C4A—H4A	108.5	C11B—C12B—H12D	110.4
C5A—C4A—H4A	108.5	H12C—C12B—H12D	108.6
C3A—C4A—H4B	108.5	C5C—B1C—C1C	105.87 (10)
C5A—C4A—H4B	108.5	C5C—B1C—N1C	110.05 (9)
H4A—C4A—H4B	107.5	C1C—B1C—N1C	110.56 (10)
C4A—C5A—C6A	112.81 (10)	C5C—B1C—H1F	114.0 (8)
C4A—C5A—B1A	110.98 (10)	C1C—B1C—H1F	113.7 (8)
C6A—C5A—B1A	107.77 (9)	N1C—B1C—H1F	102.8 (8)
C4A—C5A—H5A	108.4	C9C—N1C—C12C	105.72 (10)
C6A—C5A—H5A	108.4	C9C—N1C—B1C	114.81 (11)
B1A—C5A—H5A	108.4	C12C—N1C—B1C	113.99 (10)
C7A—C6A—C5A	115.09 (10)	C9C—N1C—H3N	107.2 (11)
C7A—C6A—H6A	108.5	C12C—N1C—H3N	107.3 (11)
C5A—C6A—H6A	108.5	B1C—N1C—H3N	107.4 (11)
C7A—C6A—H6B	108.5	C2C—C1C—C8C	113.47 (11)
C5A—C6A—H6B	108.5	C2C—C1C—B1C	110.27 (10)
H6A—C6A—H6B	107.5	C8C—C1C—B1C	107.90 (11)
C8A—C7A—C6A	115.00 (10)	C2C—C1C—H1C	108.4
C8A—C7A—H7A	108.5	C8C—C1C—H1C	108.4
C6A—C7A—H7A	108.5	B1C—C1C—H1C	108.4
C8A—C7A—H7B	108.5	C3C—C2C—C1C	115.29 (11)
C6A—C7A—H7B	108.5	C3C—C2C—H2E	108.5
H7A—C7A—H7B	107.5	C1C—C2C—H2E	108.5
C7A—C8A—C1A	115.62 (10)	C3C—C2C—H2F	108.5
C7A—C8A—H8A	108.4	C1C—C2C—H2F	108.5
C1A—C8A—H8A	108.4	H2E—C2C—H2F	107.5
C7A—C8A—H8B	108.4	C2C—C3C—C4C	114.57 (11)
C1A—C8A—H8B	108.4	C2C—C3C—H3E	108.6
H8A—C8A—H8B	107.4	C4C—C3C—H3E	108.6
N1A—C9A—C10A	105.05 (10)	C2C—C3C—H3F	108.6
N1A—C9A—H9A	110.7	C4C—C3C—H3F	108.6
C10A—C9A—H9A	110.7	H3E—C3C—H3F	107.6
N1A—C9A—H9B	110.7	C3C—C4C—C5C	115.27 (10)
C10A—C9A—H9B	110.7	C3C—C4C—H4E	108.5
H9A—C9A—H9B	108.8	C5C—C4C—H4E	108.5
C9A—C10A—C11A	101.71 (11)	C3C—C4C—H4F	108.5
C9A—C10A—H10A	111.4	C5C—C4C—H4F	108.5
C11A—C10A—H10A	111.4	H4E—C4C—H4F	107.5
C9A—C10A—H10B	111.4	C4C—C5C—C6C	113.07 (10)
C11A—C10A—H10B	111.4	C4C—C5C—B1C	111.44 (10)
H10A—C10A—H10B	109.3	C6C—C5C—B1C	107.34 (10)
C12A—C11A—C10A	104.56 (11)	C4C—C5C—H5C	108.3
C12A—C11A—H11A	110.8	C6C—C5C—H5C	108.3
C10A—C11A—H11A	110.8	B1C—C5C—H5C	108.3
C12A—C11A—H11B	110.8	C7C—C6C—C5C	114.83 (11)
C10A—C11A—H11B	110.8	C7C—C6C—H6E	108.6
H11A—C11A—H11B	108.9	C5C—C6C—H6E	108.6
N1A—C12A—C11A	106.15 (11)	C7C—C6C—H6F	108.6
N1A—C12A—H12A	110.5	C5C—C6C—H6F	108.6
C11A—C12A—H12A	110.5	H6E—C6C—H6F	107.5
N1A—C12A—H12B	110.5	C8C—C7C—C6C	114.04 (11)
C11A—C12A—H12B	110.5	C8C—C7C—H7E	108.7
H12A—C12A—H12B	108.7	C6C—C7C—H7E	108.7
C5B—B1B—C1B	106.44 (9)	C8C—C7C—H7F	108.7
C5B—B1B—N1B	110.51 (9)	C6C—C7C—H7F	108.7
C1B—B1B—N1B	111.52 (9)	H7E—C7C—H7F	107.6
C5B—B1B—H1E	113.3 (8)	C7C—C8C—C1C	115.40 (11)
C1B—B1B—H1E	112.9 (7)	C7C—C8C—H8E	108.4
N1B—B1B—H1E	102.3 (7)	C1C—C8C—H8E	108.4
C9B—N1B—C12B	105.84 (9)	C7C—C8C—H8F	108.4
C9B—N1B—B1B	114.84 (9)	C1C—C8C—H8F	108.4
C12B—N1B—B1B	113.61 (9)	H8E—C8C—H8F	107.5
C9B—N1B—H2N	105.5 (9)	C10C—C9C—N1C	106.46 (12)
C12B—N1B—H2N	107.1 (9)	C10C—C9C—H9E	110.4
B1B—N1B—H2N	109.3 (9)	N1C—C9C—H9E	110.4
C2B—C1B—C8B	113.32 (10)	C10C—C9C—H9F	110.4
C2B—C1B—B1B	110.65 (9)	N1C—C9C—H9F	110.4
C8B—C1B—B1B	107.61 (9)	H9E—C9C—H9F	108.6
C2B—C1B—H1B	108.4	C11D—C10C—C9C	108.9 (2)
C8B—C1B—H1B	108.4	C9C—C10C—C11C	100.14 (14)
B1B—C1B—H1B	108.4	C9C—C10C—H10E	111.7
C3B—C2B—C1B	114.31 (10)	C11C—C10C—H10E	111.7
C3B—C2B—H2C	108.7	C9C—C10C—H10F	111.7
C1B—C2B—H2C	108.7	C11C—C10C—H10F	111.7
C3B—C2B—H2D	108.7	H10E—C10C—H10F	109.5
C1B—C2B—H2D	108.7	C11D—C10C—H10G	109.9
H2C—C2B—H2D	107.6	C9C—C10C—H10G	109.9
C2B—C3B—C4B	114.03 (9)	C11D—C10C—H10H	109.9
C2B—C3B—H3C	108.7	C9C—C10C—H10H	109.9
C4B—C3B—H3C	108.7	H10G—C10C—H10H	108.3
C2B—C3B—H3D	108.7	C12C—C11C—C10C	100.65 (19)
C4B—C3B—H3D	108.7	C12C—C11C—H11E	111.6
H3C—C3B—H3D	107.6	C10C—C11C—H11E	111.6
C3B—C4B—C5B	115.65 (10)	C12C—C11C—H11F	111.6
C3B—C4B—H4C	108.4	C10C—C11C—H11F	111.6
C5B—C4B—H4C	108.4	H11E—C11C—H11F	109.4
C3B—C4B—H4D	108.4	C10C—C11D—C12C	112.8 (4)
C5B—C4B—H4D	108.4	C10C—C11D—H11G	109.0
H4C—C4B—H4D	107.4	C12C—C11D—H11G	109.0
C6B—C5B—C4B	113.19 (9)	C10C—C11D—H11H	109.0
C6B—C5B—B1B	107.20 (9)	C12C—C11D—H11H	109.0
C4B—C5B—B1B	111.05 (9)	H11G—C11D—H11H	107.8
C6B—C5B—H5B	108.4	C11C—C12C—N1C	107.17 (13)
C4B—C5B—H5B	108.4	N1C—C12C—C11D	102.0 (2)
B1B—C5B—H5B	108.4	C11C—C12C—H12E	110.3
C7B—C6B—C5B	115.05 (10)	N1C—C12C—H12E	110.3
C7B—C6B—H6C	108.5	C11C—C12C—H12F	110.3
C5B—C6B—H6C	108.5	N1C—C12C—H12F	110.3
C7B—C6B—H6D	108.5	H12E—C12C—H12F	108.5
C5B—C6B—H6D	108.5	N1C—C12C—H12G	111.4
H6C—C6B—H6D	107.5	C11D—C12C—H12G	111.4
C6B—C7B—C8B	115.14 (10)	N1C—C12C—H12H	111.4
C6B—C7B—H7C	108.5	C11D—C12C—H12H	111.4
C8B—C7B—H7C	108.5	H12G—C12C—H12H	109.2
			
C5A—B1A—N1A—C9A	171.27 (10)	C4B—C5B—C6B—C7B	−67.50 (14)
C1A—B1A—N1A—C9A	53.73 (13)	B1B—C5B—C6B—C7B	55.30 (13)
C5A—B1A—N1A—C12A	−65.85 (12)	C5B—C6B—C7B—C8B	−41.75 (15)
C1A—B1A—N1A—C12A	176.61 (9)	C6B—C7B—C8B—C1B	40.06 (15)
C5A—B1A—C1A—C8A	64.51 (11)	C2B—C1B—C8B—C7B	70.93 (14)
N1A—B1A—C1A—C8A	−175.66 (9)	B1B—C1B—C8B—C7B	−51.74 (13)
C5A—B1A—C1A—C2A	−59.35 (12)	C12B—N1B—C9B—C10B	29.66 (12)
N1A—B1A—C1A—C2A	60.48 (12)	B1B—N1B—C9B—C10B	155.83 (10)
C8A—C1A—C2A—C3A	−67.16 (14)	N1B—C9B—C10B—C11B	−40.54 (13)
B1A—C1A—C2A—C3A	53.49 (14)	C9B—C10B—C11B—C12B	35.66 (13)
C1A—C2A—C3A—C4A	−44.22 (15)	C9B—N1B—C12B—C11B	−7.11 (12)
C2A—C3A—C4A—C5A	43.60 (15)	B1B—N1B—C12B—C11B	−134.02 (10)
C3A—C4A—C5A—C6A	68.50 (14)	C10B—C11B—C12B—N1B	−17.95 (13)
C3A—C4A—C5A—B1A	−52.55 (14)	C5C—B1C—N1C—C9C	170.23 (11)
C1A—B1A—C5A—C4A	58.97 (12)	C1C—B1C—N1C—C9C	53.62 (14)
N1A—B1A—C5A—C4A	−61.21 (12)	C5C—B1C—N1C—C12C	−67.60 (13)
C1A—B1A—C5A—C6A	−65.01 (11)	C1C—B1C—N1C—C12C	175.79 (11)
N1A—B1A—C5A—C6A	174.81 (9)	C5C—B1C—C1C—C2C	−60.16 (13)
C4A—C5A—C6A—C7A	−68.70 (14)	N1C—B1C—C1C—C2C	59.01 (14)
B1A—C5A—C6A—C7A	54.17 (13)	C5C—B1C—C1C—C8C	64.27 (13)
C5A—C6A—C7A—C8A	−42.02 (15)	N1C—B1C—C1C—C8C	−176.56 (10)
C6A—C7A—C8A—C1A	41.88 (15)	C8C—C1C—C2C—C3C	−66.64 (15)
C2A—C1A—C8A—C7A	68.79 (14)	B1C—C1C—C2C—C3C	54.52 (15)
B1A—C1A—C8A—C7A	−53.52 (13)	C1C—C2C—C3C—C4C	−43.87 (16)
C12A—N1A—C9A—C10A	25.89 (13)	C2C—C3C—C4C—C5C	41.93 (16)
B1A—N1A—C9A—C10A	152.87 (10)	C3C—C4C—C5C—C6C	69.78 (14)
N1A—C9A—C10A—C11A	−39.37 (14)	C3C—C4C—C5C—B1C	−51.24 (14)
C9A—C10A—C11A—C12A	37.79 (14)	C1C—B1C—C5C—C4C	58.88 (12)
C9A—N1A—C12A—C11A	−1.97 (13)	N1C—B1C—C5C—C4C	−60.62 (12)
B1A—N1A—C12A—C11A	−129.45 (11)	C1C—B1C—C5C—C6C	−65.42 (12)
C10A—C11A—C12A—N1A	−22.52 (14)	N1C—B1C—C5C—C6C	175.08 (10)
C5B—B1B—N1B—C9B	171.79 (9)	C4C—C5C—C6C—C7C	−66.79 (15)
C1B—B1B—N1B—C9B	53.61 (12)	B1C—C5C—C6C—C7C	56.53 (14)
C5B—B1B—N1B—C12B	−66.17 (11)	C5C—C6C—C7C—C8C	−44.76 (16)
C1B—B1B—N1B—C12B	175.65 (9)	C6C—C7C—C8C—C1C	43.55 (17)
C5B—B1B—C1B—C2B	−59.92 (12)	C2C—C1C—C8C—C7C	68.32 (16)
N1B—B1B—C1B—C2B	60.68 (12)	B1C—C1C—C8C—C7C	−54.17 (15)
C5B—B1B—C1B—C8B	64.37 (11)	C12C—N1C—C9C—C10C	16.44 (17)
N1B—B1B—C1B—C8B	−175.03 (9)	B1C—N1C—C9C—C10C	142.99 (13)
C8B—C1B—C2B—C3B	−65.74 (13)	N1C—C9C—C10C—C11D	−5.6 (5)
B1B—C1B—C2B—C3B	55.23 (13)	N1C—C9C—C10C—C11C	−36.84 (18)
C1B—C2B—C3B—C4B	−45.59 (14)	C9C—C10C—C11C—C12C	43.9 (2)
C2B—C3B—C4B—C5B	43.93 (14)	C9C—C10C—C11D—C12C	−7.8 (8)
C3B—C4B—C5B—C6B	69.13 (13)	C10C—C11C—C12C—N1C	−35.1 (2)
C3B—C4B—C5B—B1B	−51.51 (13)	C9C—N1C—C12C—C11C	13.2 (2)
C1B—B1B—C5B—C6B	−66.31 (11)	B1C—N1C—C12C—C11C	−113.8 (2)
N1B—B1B—C5B—C6B	172.44 (9)	C9C—N1C—C12C—C11D	−19.7 (4)
C1B—B1B—C5B—C4B	57.81 (11)	B1C—N1C—C12C—C11D	−146.8 (4)
N1B—B1B—C5B—C4B	−63.44 (11)	C10C—C11D—C12C—N1C	17.7 (8)
